# Treatment of cervical insufficiency and/or a short cervix with antimicrobial agents can restore cervical length and lead to pregnancy prolongation and term delivery

**DOI:** 10.1080/14767058.2024.2349789

**Published:** 2024-05-15

**Authors:** Roberto Romero, Tinnakorn Chaiworapongsa, Arun Meyyazhagan, Eunjung Jung, Bo Hyun Yoon, David Kmak, Lami Yeo, Jeffrey Johnson, Robert Para, Chaur-Dong Hsu

**Affiliations:** Pregnancy Research Branch,NICHD/NIH/DHHS, Bethesda,MD, USA; Department of Obstetrics and Gynecology, Wayne State University School of Medicine, Detroit, MI, USA; Pregnancy Research Branch,NICHD/NIH/DHHS, Bethesda, MD, USA; Department of Obstetrics and Gynecology, Busan Paik Hospital, Inje University College of Medicine, Busan, Republic of Korea; Biomedical Research Institute, Seoul National University, Hospital, Seoul, Republic of Korea; Department of Obstetrics and Gynecology, Wayne State University School of Medicine, Detroit, MI, USA; Department of Obstetrics and Gynecology, Wayne State University School of Medicine, Detroit, MI, USA; Division Director of Maternal-Fetal Medicine, Mount Carmel, Health System, Columbus, OH, USA; Advocate Aurora Health, Milwaukee, WI, USA; Department of Obstetrics and Gynecology, College of Medicine Tucson, University of Arizona, Tucson, AZ, USA

## Dear Editor

We thank Dr. Kosińska-Kaczyńska et al. for their interest in our report [1] (see Video in the supplementary material) and for sharing the details of a patient with a sonographic short cervix at 31 weeks presenting with what many clinicians would diagnose as cervical insufficiency (dilated cervix and visible membranes as shown in Figure 1B) treated with antimicrobial agents and vaginal progesterone. Our case and that presented in the Letter to the Editor [2] highlight several points: (1) cervical insufficiency is generally considered to be a condition diagnosed in the second trimester [3]; however, it can present in the third; (2) a sonographic short cervix can precede the development of full-blown cervical insufficiency [1], both of which are not discrete conditions but syndromes caused by multiple pathologic processes such as intra-amniotic infection [3–8], sterile intra-amniotic inflammation [9], and a decline in progesterone action [3,10]; (3) antimicrobial treatment with a combination of three antibiotics has been shown to be effective in eradicating intra-amniotic inflammation (with and without intra-amniotic infection) in patients with preterm labor and intact membranes [11], preterm prelabor rupture of the membranes (PROM) [12,13], cervical insufficiency [14] in the mid-trimester, and threatened mid-trimester miscarriage [15]. Dr. Kosińska-Kaczyńska et al. suggest that antimicrobial agents may be an option for patients who are not candidates for cerclage because they present in the third trimester [2]; (4) amniotic fluid analysis remains the only way to diagnose intra-amniotic infection and intra-amniotic inflammation accurately and to monitor response to therapy; (5) non-invasive biomarkers of intra-amniotic inflammation are urgently needed to assess the likelihood of intra-amniotic inflammation, and there is evidence that analysis of vaginal fluid for biomarkers such as interleukin-6, fetal fibronectin, and maternal blood (e.g. C-reactive protein) can be helpful in the identification of the patient at risk [16,17]. This will help counsel patients about the potential benefits of amniocentesis; (6) previous observations suggest that antimicrobial agents may be effective in eradicating amniotic fluid sludge [18]. In most cases of infection-related sludge, the organisms are likely to be *Ureaplasma* species or other bacteria treated with the combination of ceftriaxone, clarithromycin, and metronidazole. It is important to remember that some cases can be due to fungi [18,19], which would not be treated with this combination, and that other cases may not be due to infection or inflammation but rather intra-amniotic bleeding [20]; (7) the duration of therapy is best monitored by following biomarkers rather than empiric treatment [11–13,21]; (8) randomized clinical trials in these conditions (acute cervical insufficiency with and without sludge in the second and third trimesters) are optimal but difficult to conduct because it would require randomization of patients to expectant management [22]; (9) the efficacy of vaginal progesterone in preventing preterm delivery has been persuasively proven in patients with a short cervix in the second trimester [23,24]. Whether this intervention is efficacious after 24 weeks remains an important clinical question [25,26]; and (10) treatment aimed at the specific causes of obstetrical syndromes (i.e. etiology-based treatment) is required to improve pregnancy outcome and to make progress in obstetrics [27]. Clinical signs and symptoms and demographic characteristics are insufficient to diagnose the causes of obstetrical syndromes. Hence, we believe that patients with preterm labor and intact membranes, preterm PROM, and cervical insufficiency are best managed by specialists in maternal-fetal medicine with expertise in ultrasound and amniocentesis and with in-depth knowledge of the biology of infection and inflammation.

## Supplementary Material

Supplementary Material

## Data Availability

Data sharing is not applicable as no new data were created or analyzed in this article.
